# Biofilm assembly becomes crystal clear – filamentous bacteriophage organize the *Pseudomonas aeruginosa* biofilm matrix into a liquid crystal

**DOI:** 10.15698/mic2016.01.475

**Published:** 2015-12-31

**Authors:** Patrick R. Secor, Laura K. Jennings, Lia A. Michaels, Johanna M. Sweere, Pradeep K. Singh, William C. Parks, Paul L. Bollyky

**Affiliations:** 1 Department of Microbiology, University of Washington, Seattle, WA, USA.; 2 Department of Medicine, Stanford University, Stanford, CA, USA.; 3 Department of Medicine, Cedars-Sinai Medical Center, Los Angeles, CA, USA.

**Keywords:** biofilm, Pseudomonas aeruginosa, filamentous bacteriophage, chronic infection, cystic fibrosis, liquid crystal, soft matter physics

## Abstract

*Pseudomonas aeruginosa* is an opportunistic bacterial pathogen associated with many types of chronic infection. At sites of chronic infection, such as the airways of people with cystic fibrosis (CF), *P. aeruginosa* forms biofilm-like aggregates. These are clusters of bacterial cells encased in a polymer-rich matrix that shields bacteria from environmental stresses and antibiotic treatment. When *P. aeruginosa* forms a biofilm, large amounts of filamentous Pf bacteriophage (phage) are produced. Unlike most phage that typically lyse and kill their bacterial hosts, filamentous phage of the genus Inovirus, which includes Pf phage, often do not, and instead are continuously extruded from the bacteria. Here, we discuss the implications of the accumulation of filamentous Pf phage in the biofilm matrix, where they interact with matrix polymers to organize the biofilm into a highly ordered liquid crystal. This structural configuration promotes bacterial adhesion, desiccation survival, and antibiotic tolerance – all features typically associated with biofilms. We propose that Pf phage make structural contributions to *P. aeruginosa* biofilms and that this constitutes a novel form of symbiosis between bacteria and bacteriophage.

## Filamentous bacteriophage in *P. aeruginosa *biofilms

When *P. aeruginosa* forms a biofilm, Pf phages are produced in abundance. This has been observed by several laboratories using different methods to grow biofilms (flow cells, chemostats, etc.). In addition to being produced under biofilm conditions *in vitro*, Pf prophages are also prevalent amongst clinical *P. aeruginosa* CF isolates. Indeed, we were able to detect intact Pf phage in CF sputum at concentrations averaging 10^7^/ml.

## Filamentous bacteriophage and liquid crystal assembly 

**Figure 1:**
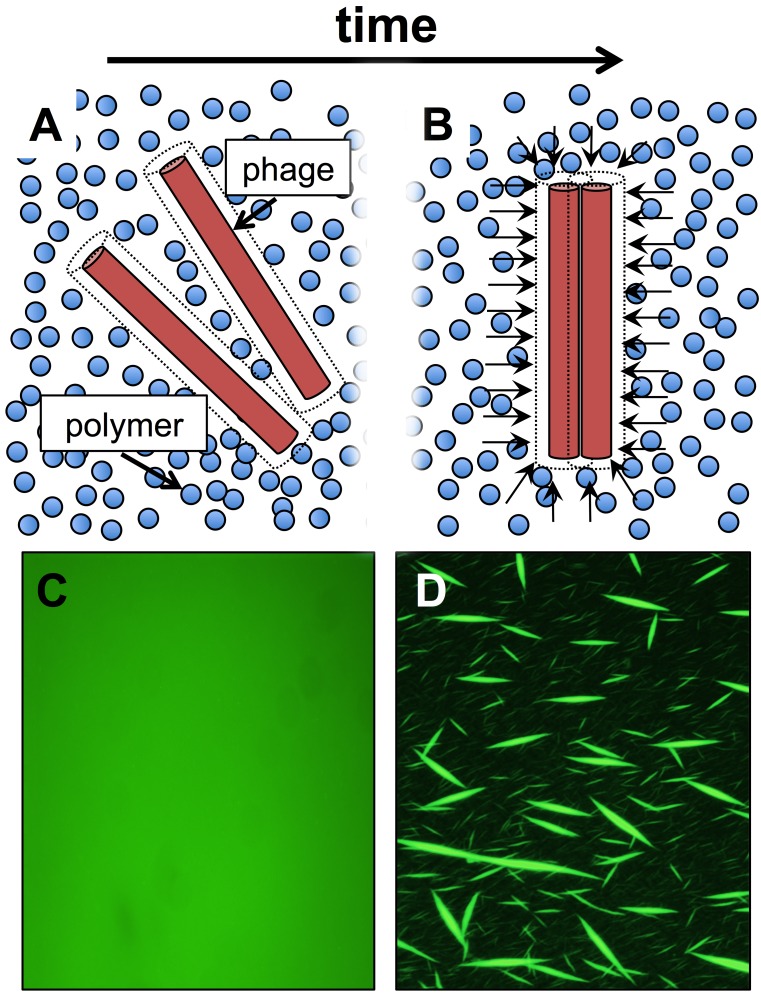
FIGURE 1: Schematic of how depletion attraction drives liquid crystal assembly from Pf phage within polymer-rich environments. **(A)** Dashed lines around phage particles represent an exclusion volume not accessible to the polymer coil’s center of mass. When phages are dispersed, the volume inaccessible to the polymers is maximized. **(B)** When phages come into close proximity to each other, their exclusion volumes overlap, increasing the total volume accessible to the polymers. This maximizes the entropy of the system. Arrows represent an osmotic imbalance that polymers exert on the liquid crystalline phage bundle. This osmotic pressure is what physically holds the liquid crystal together. **(C)** Fluorescently labeled Pf phages (green) are initially dispersed when mixed with the host polymer hyaluronan. **(D)** As time progresses, the phages form liquid crystalline structures.

It is well established that filamentous Inoviruses, including Pf phage, can assemble liquid crystals – a state of matter between that of a liquid and a solid. Indeed, due to their uniform (monodisperse) diameter and length, Inoviruses have been used for years as an experimental model system to study the physics of liquid crystals. High concentrations of filamentous phage will spontaneously align and form a liquid crystal, owing to steric forces between neighboring phage particles. Non-adsorbing polymers can potentiate this liquid crystal assembly through a crowding mechanism called depletion attraction. This is an entropic force that causes the phage to segregate from the polymers, resulting in an enhanced propensity to form liquid crystals (Figure 1). 

## The biophysical properties of Pf phage transform the biofilm matrix into a liquid crystal

**Figure 2:**
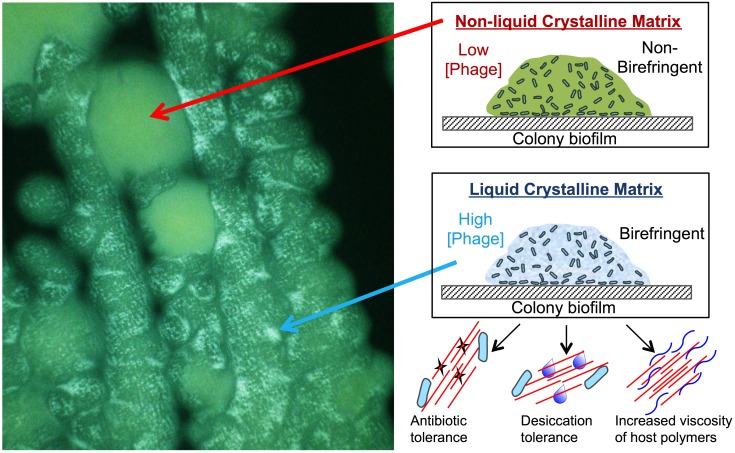
FIGURE 2: Pf phages organize the *P. aeruginosa* biofilm matrix into a liquid crystal. The birefringence of the liquid crystalline biofilm matrix can be visualized by placing colony biofilms on an agar pad between crossed polarizing lenses. The liquid crystalline matrix can change the polarization of light allowing it to pass through both polarizing lenses. Thus, non-liquid crystalline biofilms appear opaque (red arrow) while liquid crystalline biofilms appear bright (blue arrow). The organization of the biofilm matrix into a liquid crystal enhances adhesion, antibiotic tolerance, and desiccation survival. Further, Pf phage can increase the viscosity of host polymers such as mucin and DNA.

Given its inherently crowded, polymer-rich nature, the biofilm matrix offers an ideal environment for depletion attraction to organize filamentous phage into liquid crystalline structures. We quantified the liquid crystalline properties of *P. aeruginosa* biofilms by taking advantage of the optical properties of liquid crystals. In particular, they exhibit birefringence, or double refraction, whereby incoming light is split into two beams with perpendicular polarization. Thus, birefringence is a direct measurement of molecular order. Using this technique in conjunction with studies that included Pf phage deficient and replete *P. aeruginosa *strains, we found that Pf phage were both necessary and sufficient to assemble *P. aeruginosa* biofilms into birefringent liquid crystals (Figure 2).

## Pf phage and a liquid crystalline matrix enhance the pathogenic features of *P. aeruginosa* biofilms

The crystalline structure of the matrix enhanced biofilm fitness in several ways. One function of the biofilm matrix is to offer bacteria protection from desiccation. We found that bacteria within a liquid crystalline matrix more efficiently retained moisture and were better able to survive desiccation than bacteria within an unordered matrix. This enhanced ability to withstand desiccation may be due to the inherent viscosity of liquid crystals, as viscous materials generally display low rates of evaporation. It is interesting to note that epidemic strains of *P. aeruginosa,* such as the Liverpool epidemic strain, contain Pf prophage. Given that desiccation tolerance is important for the transmission of bacteria from the environment to a host, Pf phage may enhance the desiccation survival of epidemic strains, contributing to their transmissibility.

We also found that a crystalline biofilm matrix protects bacteria from killing by antibiotics. Bacteria within a liquid crystalline matrix were more tolerant to aminoglycosides such as tobramycin. Aminoglycosides are cationic antibiotics; therefore we hypothesized that they would be sequestered by anionic Pf phage in the biofilm matrix, reducing bacterial killing. This was indeed the case. Moreover, liquid crystalline phases of Pf phage were able to bind tobramycin more efficiently than disordered Pf phage. While the mechanism behind this enhanced binding is still unclear, we speculate that bundles of negatively charged Pf phage within the liquid crystalline biofilm matrix would create a dense, localized negative charge that could attract and sequester charged antibiotics. One prediction of this model is that antibiotics with a net neutral charge, like ciprofloxacin, would not be sequestered by Pf phage. Indeed, bacteria within a crystalline matrix were equally sensitive to ciprofloxacin relative to bacteria in a non-crystalline matrix. Together, these data support a role for Pf phage in tolerance to aminoglycosides and potentially other cationic antimicrobials, such as antimicrobial peptides.

## Host polymers and Pf bacteriophage

In CF, viscous airway secretions containing mucin, DNA, F-actin, hyaluronan, and other polymers accumulate in the airways, contributing to pathogenesis. We found that in addition to bacterial polymers, Pf phage likewise assemble liquid crystals in the presence of host polymers, causing an increase in viscosity. This ability of Pf phage to increase the viscosity of the local environment could trap bacteria at sites of infection, reducing bacterial invasiveness, thereby promoting a chronic, localized infection.

## Conclusions

Our findings highlight a previously unknown role for filamentous Pf phage in organizing the *P. aeruginosa* biofilm matrix into a liquid crystalline structure. These findings help ground our understanding of biofilm formation within established paradigms of soft matter physics.

We demonstrated that Pf phage contribute to several bacterial phenotypes related to chronic infection as a result of these structural contributions to the *P. aeruginosa* biofilm matrix (Figure 2). In the context of CF, Pf phage may allow *P. aeruginosa* to resist desiccation outside of the body, which could potentially increase the chances that it can be transmitted to a host and establish chronic airway infections. Once *P. aeruginosa* is established within the airways of infected individuals, Pf phage may increase the viscosity of CF airway secretions, and potentially contribute to bacterial colonization and airway obstruction. As the infection becomes further established, the liquid crystalline biofilm matrix could enhance antimicrobial tolerance, further promoting infection persistence. 

In light of these potential contributions to chronic *P. aeruginosa* infection, targeting the production of filamentous phage or the liquid crystalline organization of the biofilm matrix may offer alternative therapeutic approaches. Conceivably, these could be directed at disrupting biofilm assembly, dispersing pre-existing biofilms, or increasing the susceptibility of bacteria to antimicrobials.

It is tempting to speculate that filamentous phage may likewise organize biofilms formed by other bacterial species into liquid crystalline structures. Inoviruses are prevalent amongst Gram-negative bacterial species (e.g., *Escherichia coli, Vibrio cholerae, *etc.) and we showed that some of these other phages likewise assemble liquid crystals. Further, in addition to filamentous phage, phage with other capsid geometries, such as those with an icosahedral head, might also influence matrix structure and function when produced in abundance. For example, depletion attraction can cause spherical particles to aggregate and form a variety of structures that link together into extended networks (i.e., they form a gel).

Finally, we propose that Pf phage may play a more symbiotic role in *P. aeruginosa* biofilms than previously suspected. The production of Pf phage has been linked to the progression of the biofilm lifecycle by promoting localized cell lysis, resulting in the accumulation of substrate (e.g. DNA) for biofilm formation. While Pf phage only induces lysis in a subpopulation of bacterial cells, our findings suggest that it may also contribute to bacterial fitness through effects on adhesion, viscosity, tolerance to desiccation, and tolerance to antibiotics.

